# Association of HLA class I and II gene polymorphisms with acetaminophen-related Stevens–Johnson syndrome with severe ocular complications in Japanese individuals

**DOI:** 10.1038/s41439-019-0082-6

**Published:** 2019-10-28

**Authors:** Mayumi Ueta, Ryosuke Nakamura, Yoshiro Saito, Katsushi Tokunaga, Chie Sotozono, Toshio Yabe, Michiko Aihara, Kayoko Matsunaga, Shigeru Kinoshita

**Affiliations:** 1 Department of Frontier Medical Science and Technology for Ophthalmology, Kyoto Prefectural University of Medicine, Kyoto, Japan; 2 Division of Medicinal Safety Science, National Institute of Health Sciences, Kawasaki, Japan; 3 Department of Human Genetics, Graduate School of Medicine, University of Tokyo, Tokyo, Japan; 4 Department of Ophthalmology, Kyoto Prefectural University of Medicine, Kyoto, Japan; 5 Tokyo Metropolitan Red Cross Blood Center, Tokyo, Japan; 6 Department of Environmental Immuno-Dermatology, Yokohama City University Graduate School of Medicine, Yokohama, Japan; 7 Department of Integrative Medical Science for Allergic Disease, Fujita Health University School of Medicine, Aichi, Japan; 8 Present Address: Genome Medical Science Project (Toyama), National Center for Global Health and Medicine, Tokyo, Japan

**Keywords:** Predictive markers, Immunological disorders

## Abstract

Stevens–Johnson syndrome (SJS) and toxic epidermal necrolysis (TEN) are acute-onset mucocutaneous diseases induced by infectious agents and/or inciting drugs. We have reported that the main causative drugs for SJS/TEN with severe ocular complications (SOC) were cold medicines, including multi-ingredient cold medications and nonsteroidal anti-inflammatory drugs (NSAIDs). Moreover, we also reported that acetaminophen is the most frequent causative drug in various cold medicines. In this study, we focused on acetaminophen-related SJS/TEN with SOC and analyzed *HLA-class II (HLA-DRB1, DQB1)* in addition to *HLA-class I (HLA-A, B, C)*. We studied the histocompatibility antigen genes *HLA-DRB1* and *DQB1* in addition to *HLA-A, B*, and *C* in 80 Japanese patients with acetaminophen-related SJS/TEN with SOC. We performed polymerase chain reaction amplification followed by hybridization with sequence-specific oligonucleotide probes (PCR-SSO) using commercial bead-based typing kits. We also used genotyped data from 113 healthy volunteers for *HLA-DRB1* and *DQB1*, and 639 healthy volunteers for *HLA-A, B, and C*. HLA-DRB1*08:03 and DRB1*12:02 were associated with acetaminophen-related SJS/TEN with SOC, although the results ceased to be significant when we corrected the *p*-value for the number of alleles detected. HLA-A*02:06 was strongly associated with acetaminophen-related SJS/TEN with SOC (carrier frequency: *p* = 4.7 × 10^−12^, Pc = 6.6 × 10^−11^, OR = 6.0; gene frequency: *p* = 8.0 × 10^−13^, Pc = 1.1 × 10^−11^, OR = 4.9). HLA-B*13:01 (carrier frequency: *p* = 2.0 × 10^−3^, Pc = 0.042, OR = 4.1; gene frequency: *p* = 2.2 × 10^−3^, Pc = 0.047, OR = 3.9), HLA-B*44:03 (carrier frequency: *p* = 2.1 × 10^−3^, Pc = 0.045, OR = 2.4) and HLA-C*14:03 (carrier frequency: *p* = 3.4 × 10^−3^, Pc = 0.045, OR = 2.3) were also significantly associated, while HLA-A*24:02 was inversely associated (gene frequency: *p* = 6.3 × 10^−4^, Pc = 8.8 × 10^−3^, OR = 0.5). Acetaminophen-related SJS/TEN with SOC was not associated with *HLA-class II (HLA-DRB1, DQB1)*. However, for acetaminophen-related SJS/TEN with SOC, we found an association with HLA-B*13:01 and HLA- C*14:03 in addition to HLA-A*02:06 and HLA-B*44:03, which have been described previously.

## Stevens–Johnson syndrome: Gene variants predispose to reaction to cold drug

Researchers have identified two new gene variants that could predispose to a rare drug reaction to a common cold drug. Stevens–Johnson syndrome can be triggered by drugs and infections and is characterized by blistering of the skin and mucous membranes. Mayumi Ueta of Kyoto Prefectural University of Medicine in Japan and colleagues found an association between variants in two genes coding for human leukocyte antigen (HLA) class I molecules and Stevens–Johnson syndrome induced by acetominophen and complicated by severe eye ulcerations in Japanese patients. HLA genes code for cell surface proteins that help the immune system distinguish normal from foreign cells. Other studies have identified population-specific HLA variants associated with the syndrome. This study provides further evidence of HLA-specific ethnic differences in people diagnosed with the condition.

## Introduction

Stevens–Johnson syndrome (SJS) is an acute inflammatory vesiculobullous reaction of the skin and the mucosa of the ocular surface, oral cavity, and genitals. Patients with extensive skin detachment and a poor prognosis have toxic epidermal necrolysis (TEN).

In the acute stage of SJS/TEN, approximately 50% of patients present with severe ocular lesions, such as severe conjunctivitis with pseudomembrane and ocular surface epithelial defects^1^.

The mortality rate of SJS/TEN is high (3% for SJS and 27% for TEN)^2^, although its reported annual incidence is only 1–6 per million individuals^1^. The extreme rarity of cutaneous and ocular surface reactions to drug therapies led us to suspect individual susceptibility.

While associations between SJS/TEN and many kinds of inciting drugs have been documented^3^, we have reported that the main causative drugs for SJS/TEN with severe ocular complications (SOC) were cold medicines; approximately 80% of our SJS/TEN with SOC patients developed SJS/TEN within several days after taking cold medicines, including multi-ingredient cold medications and nonsteroidal anti-inflammatory drugs (NSAIDs), to combat the common cold^4–7^. Moreover, we also reported that acetaminophen is the most frequent causative drug ingredient in various cold medicines and that cold medicine-related SJS/TEN with SOC, including acetaminophen-related SJS/TEN with SOC, was significantly associated with HLA-A*02:06 and HLA-B*44:03 in Japanese individuals^6^. However, in a previous study in which we focused on cold medicines, we analyzed only *HLA-class I (HLA-A, B, and C)*.

On the other hand, Power et al.^8^ reported that HLA-DQB1*06:01 was associated with Caucasian patients with ocular complications of SJS.

In this study, we focused on acetaminophen-related SJS/TEN with SOC and analyzed *HLA-class II (HLA-DRB1, DQB1)* in addition to *HLA-class I (HLA-A, B, C)*.

## Materials and methods

### Patients

Our study was approved by the institutional review boards of Kyoto Prefectural University of Medicine, Kyoto, Japan, and the National Institute of Health Sciences, Kawasaki, Japan. All experimental procedures were conducted in accordance with the principles set forth in the Helsinki Declaration and Ethical Guidelines for Human Genome/Gene Analysis Research of Japan. The purpose of the study and the experimental protocols were explained to all participants, and their prior written informed consent was obtained.

Because ophthalmologists encounter patients not only in the acute stage but also in the chronic stage, it is not easy for ophthalmologists to render a differential diagnosis of SJS or TEN when patients present in the chronic stage because the vesiculobullous skin lesions evident in the acute stage have healed by the chronic stage^5^. Thus, our ophthalmologic diagnosis of SJS/TEN was based on a confirmed history of acute-onset high fever, serious mucocutaneous illness with skin eruptions, and the involvement of at least 2 mucosal sites, including the ocular surface^4,6,7,9,10^.

SJS/TEN patients with SOC in the acute stage often suffer severe ocular sequelae such as vision loss and very severe dry eye that prevent them from having a normal life^11^. We defined acute-stage SOC as a condition with severe conjunctivitis with pseudomembrane and epithelial defects on the ocular surface (cornea and/or conjunctiva)^12^ and chronic-stage SOC as a condition with ocular sequelae such as severe dry eye, trichiasis, symblepharon, and conjunctival invasion into the cornea^11^.

For HLA genotyping, we enrolled 80 Japanese SJS/TEN with SOC patients (64 of them were recruited by Kyoto Prefectural University of Medicine, and 16 of them were recruited by the Japan Severe Adverse Reactions research group (JSAR research group, mainly operated by the National Institute of Health Sciences)). The average patient age was 38.9 ± 17.6 (SD) years, and the average onset age was 30.1 ± 16.5 (SD) years; the male:female ratio was 32:48. Note that the results of HLA class I (HLA-A, B, and C types) from 73 cases of acetaminophen-related SJSTEN patients with SOC were reported previously^6^.

### Controls

We used the genotyped data of 113 healthy volunteer blood donors for *HLA class II (DRB1, DQB1)* and of 639 healthy volunteers for *HLA class I (A, B, C)*. All volunteers were Japanese residing in Japan. They have been used in our previous study^6,9^.

### HLA genotyping

We studied the histocompatibility antigen genes *HLA-A, B, C, DRB1*, and *DQB1* of 80 Japanese acetaminophen-related SJS/TEN with SOC patients. These alleles were detected by the PCR-Luminex typing method using the WAKFlow HLA Typing Kit (Wakunaga, Hiroshima) as previously reported^6,9,13^. Genotype determination and data analysis were performed automatically using the WAKFlow typing software.

### Statistical methods

We compared the carrier frequency of individual *HLA* alleles in our patients and controls based on the dominant model using Fisher’s exact test (JMP version 11 software; SAS Institute Japan Ltd., Tokyo, Japan). Each allele was assessed as an independent variable, and separate *p*-values were calculated. A *p*-value of <0.05 was regarded as significant. In addition, the *p*-values were corrected for the number of alleles tested. The alleles with total numbers under 8 for *HLA-class II (DRB1, DQB1)* and under 21 for *HLA class I (A, B, C)* were included in others.

## Results

Table 1 shows the results on *HLA-DRB1* alleles. Although the correction of the *p*-values for the number of alleles detected (*n* = 16) rendered the result not significant, HLA-DRB1*08:03 (carrier frequency: *p* = 0.016, Pc = 0.26, OR = 2.5; gene frequency: *p* = 0.017, Pc = 0.17, OR = 2.3) and DRB1*12:02 (carrier frequency: *p* = 0.024, Pc = 0.38, OR = 3.9; gene frequency: *p* = 0.027, Pc = 0.27, OR = 3.7) were associated with acetaminophen-related SJS/TEN with SOC. There was also no association between *HLA-DQB1* and acetaminophen-related SJS/TEN with SOC (Supplementary Table 1).

**Table 1:** Association between HLA class II and acetaminophen-related SJS/TEN with SOC

HLA-DRB1	Carrier frequency					Gene Frequency				
	Case	Control	*p*-value (Fisher)	Corrected *p*-value	OR (95% CI)	Case	Control	*p*-value (Fisher)	Corrected *p*-value	OR (95% CI)
DRB1*08:03	27.5% (22/80)	13.3% (15/113)	**0.0161**	0.258	2.5 (1.2–5.2)	15.0% (24/160)	7.1% (16/226)	**0.0168**	0.167	2.3 (1.2–4.5)
DRB1*12:02	12.5% (10/80)	3.5% (4/113)	**0.0239**	0.382	3.9 (1.2–12.9)	6.3% (10/160)	1.8% (4/226)	**0.0265**	0.269	3.7 (1.1–12.0)

*OR* odds ratio, *CI* confidence interval

Bold values indicates *p* < 0.05

As shown in Table 2a, HLA-A*02:06 was strongly associated with acetaminophen-related SJS/TEN with SOC (carrier frequency: *p* = 4.7 × 10^−12^, Pc = 6.6 × 10^−11^, OR = 6.0; gene frequency: *p* = 8.0 × 10^−13^, Pc = 1.1 × 10^−11^, OR = 4.9). HLA-A*24:02 was inversely associated (carrier frequency: *p* = 5.3 × 10^−3^, Pc = 0.074, OR = 0.5; gene frequency: *p* = 6.3 × 10^−4^, Pc = 8.8 × 10^−3^, OR = 0.5). HLA-A*33:03 was also associated (carrier frequency: *p* = 0.011, Pc = 0.16, OR = 2.1; gene frequency: *p* = 0.12, Pc = 0.16, OR = 2.0), and HLA-A*11:01 (carrier frequency: *p* = 0.024, Pc = 0.34, OR = 0.4; gene frequency: *p* = 0.037, Pc = 0.52, OR = 0.4) and A*26:01 (gene frequency: *p* = 0.047, Pc = 0.65, OR = 0.4) were inversely associated, but the association ceased to be significant when we corrected the *p*-value for the number of alleles detected (*n* = 14).

**Table 2:** Association between HLA class I and acetaminophen-related SJS/TEN with SOC

	Carrier frequency					Gene frequency				
	Case	Control	*p*-value (Fisher)	corrected *p*-value	OR (95% CI)	Case	Control	*p*-value (Fisher)	corrected *p-*value	OR (95% CI)
a										
HLA-A										
A*02:06	48.8% (39/80)	13.62% (87/639)	**4.71E−12**	**6.59E−11**	6.0 (3.7–9.9)	27.5% (44/160)	7.12% (91/1278)	**8.01E−13**	**1.12E−11**	4.9 (3.3–7.4)
A*11:01	7.5% (6/80)	17.21% (110/639)	**0.0239**	0.335	0.4 (0.2–0.9)	4.4% (7/160)	9.31% (119/1278)	**0.0372**	0.521	0.4 (0.2–1.0)
A*24:02	43.8% (35/80)	60.72% (388/639)	**5.31E−3**	0.074	0.5 (0.3–0.8)	23.8% (38/160)	37.32% (477/1278)	**6.25E−4**	**8.75E−3**	0.5 (0.4–0.8)
A*26:01	6.3% (5/80)	14.40% (92/639)	0.0543		0.4 (0.2–1.0)	3.1% (5/160)	7.43% (95/1278)	**0.0465**	0.651	0.4 (0.2–1.0)
A*33:03	25.0% (20/80)	13.46% (86/639)	**0.011**	0.155	2.1 (1.2–3.7)	13.1% (21/160)	7.12% (91/1278)	**0.0117**	0.163	2.0 (1.2–3.3)
b										
HLA-B										
B*13:01	11.3% (9/80)	2.97% (19/639)	**1.98E−3**	**0.0415**	4.1 (1.8–9.5)	5.6% (9/160)	1.49% (19/1278)	**2.23E−3**	**0.0468**	3.9 (1.8–8.9)
B*15:01	5.0% (4/80)	16.90% (108/639)	**4.77E−3**	0.100	0.3 (0.1–0.7)	2.5% (4/160)	8.61% (110/1278)	**4.68E−3**	0.982	0.3 (0.1–0.7)
B*44:03	30.0% (24/80)	15.02% (96/639)	**2.12E−3**	**0.0446**	2.4 (1.4–4.1)	15.0% (24/160)	7.67% (98/1278)	**3.74E−3**	0.0784	2.1 (1.3–3.4)
B*46:01	17.5% (14/80)	8.76% (56/639)	**0.0251**	0.527	2.2 (1.2–4.2)	8.8% (14/160)	4.46% (57/1278)	**0.0304**	0.640	2.1 (1.1–3.8)
B*52:01	8.8% (7/80)	19.87% (127/639)	**0.0144**	0.303	0.4 (0.2–0.9)	4.4% (7/160)	10.02% (128/1278)	**0.0205**	0.431	0.4 (0.2–0.9)
c										
HLA-C										
C*03:04	32.5% (26/80)	22.07% (141/639)	**0.0484**	0.629	1.7 (1.0–2.8)	16.9% (27/160)	11.89% (152/1278)	0.0759		1.5 (1.0–2.4)
C*12:02	8.8% (7/80)	19.87% (127/639)	**0.0144**	0.188	0.4 (0.2–0.9)	4.4% (7/160)	10.02% (128/1278)	**0.0205**	0.267	0.4 (0.2–0.9)
C*14:03	28.8% (23/80)	14.87% (95/639)	**3.44E−3**	**0.0447**	2.3 (1.4–3.9)	14.4% (23/160)	7.67% (98/1278)	**6.34E−3**	0.0825	2.0 (1.2–3.3)

*OR* odds ratio, *CI* confidence interval

Bold values indicates *p* < 0.05

Table 2b shows the results on *HLA-B alleles*. B*13:01 (carrier frequency: *p* = 2.0 × 10^−3^, Pc = 0.042, OR = 4.1; gene frequency: *p* = 2.2 × 10^−3^, Pc = 0.047, OR = 3.9), and HLA-B*44:03 (carrier frequency: *p* = 2.1 × 10^−3^, Pc = 0.045, OR = 2.4; gene frequency: *p* = 3.7 × 10^−3^, Pc = 0.078, OR = 2.1) was significantly associated. HLA-B*46:01 was also associated (carrier frequency: *p* = 0.025, Pc = 0.53, OR = 2.2; gene frequency: *p* = 0.030, Pc = 0.64, OR = 2.1), and HLA-B*15:01 (carrier frequency: *p* = 4.8 × 10^−3^, Pc = 0.10, OR = 0.3; gene frequency: *p* = 4.7 × 10^−3^, Pc = 0.98, OR = 0.3) and B*52:01 (carrier frequency: *p* = 0.014, Pc = 0.30, OR = 0.4; gene frequency: *p* = 0.021, Pc = 0.43, OR = 0.4) were inversely associated, although the results ceased to be significant when we corrected the *p*-value for the number of alleles detected (*n* = 21).

Table 2c shows the results for *HLA-C alleles*. HLA-C*14:03 (carrier frequency: *p* = 3.4 × 10^−3^, Pc = 0.045, OR = 2.3; gene frequency: *p* = 6.3 × 10^−3^, Pc = 0.083, OR = 2.0) was significantly associated. HLA-C*03:04 was also associated (carrier frequency: *p* = 0.048, Pc = 0.63, OR = 1.7), and HLA-C*12:02 was inversely associated (carrier frequency: *p* = 0.014, Pc = 0.19, OR = 0.4; gene frequency: *p* = 0.021, Pc = 0.27, OR = 0.4), although the results ceased to be significant when we corrected the *p*-value for the number of alleles detected (*n* = 13). The results of *HLA-class I (HLA-A, B, C)* are shown in Supplementary Table 2.

## Discussion

We previously reported that approximately 80% of our SJS/TEN with SOC patients developed SJS/TEN within several days after taking cold medicines, including multi-ingredient cold medications and NSAIDs, to combat the common cold^4,5,7,13^. More than half of SJS/TEN with SOC patients developed SJS after taking cold medicines in the Brazilian population^14^. Cold medicine, including NSAIDs, might be associated with SOC in SJS/TEN patients in the Korean population^15^. Moreover, 69% of SJS/TEN with SOC patients had a history of taking cold medicine before the onset of SJS/TEN in the Thai population^16^.

Acetaminophen, also called paracetamol, is the most frequent causative drug for cold medicine-related SJS/TEN with SOC in Japanese^6^ and Thai individuals^16^. On the other hand, dipyrone is the most frequent cause of cold medicine-related SJS/TEN with SOC in Brazil^14^.

In this study, we analyzed both class I and class II HLA types focused on acetaminophen-related SJS/TEN with SOC and found that there was also no association between *HLA class II (HLA-DQB1, DQB1)* and acetaminophen-related SJS/TEN with SOC and confirmed the strong association with HLA-A*02:06 and the significant association with HLA-B*44:03, as in our previous study^6^. Moreover, we also found associations with HLA-B*13:01 and HLA-C*14:03 and an inverse association with HLA-A*24:02.

Power et al.^8^ reported that HLA-DQB1*06:01 was associated with Caucasian patients with ocular complications of SJS. However, we could not detect an association between Japanese SJS/TEN and SOC and HLA-DQB1*06:01.

HLA-A*02:06 was significantly associated with Japanese^6^ and Korean^17^ cold medicine-related SJS/TEN with SOC. On the other hand, Brazilian cold medicine-related SJS/TEN with SOC was significantly associated with HLA-A*66:01 in individuals with both Pardo and European ancestry and HLA-B*44:03 and HLA-C*12:03 for European ancestry^14^. Moreover, cold medicine-related SJS/TEN with SOC in Thailand was significantly associated with the HLA-B*44:03-HLA-C*07:01 haplotype, although their main causative drug is paracetamol^16^. Thus, there are ethnic differences in associated HLAs with cold medicine-related SJS/TEN with SOC.

Acetaminophen-associated HLA type might be quite similar to HLA associated with cold medicine-related SJS/TEN with SOC. The binding modes of acetaminophen, ibuprofen, and loxoprofen at the antigenic peptide-binding groove of the HLA-A∗02:06 molecule are different because their molecules are not similar, and their composite risk indexes are also different^18^. Thus, we suggest that the common function of cold medicines, such as acetaminophen and NSAIDs, is most important for the onset of SJS/TEN with SOC. The common function of cold medicines is a suppression function of prostaglandin E_2_ (PGE_2_) production, which suppresses mucocutaneous inflammation; PGE_2_ acts at EP3 (PGE_2_ receptor 3), which is one of the 4 receptors (EP1, EP2, EP3, EP4) for PGE_2_, in the epidermis^19^ and mucosal epithelium^10,20^, negatively regulating mucocutaneous inflammation. Cold medicine, including acetaminophen, could downregulate inflammatory suppressing mechanism(s) by PGE_2_ and might augment the abnormal immune response resulting in the induction of SJS/TEN with SOC^5,21^.

Acetaminophen (paracetamol) is thought to be a safe drug and is widely prescribed for children with cold symptoms or is widely included in commercial cold medicines. However, for SJS/TEN with SOC, acetaminophen could be the main causative drug. Therefore, physicians should prescribe it with knowledge about SJS/TEN.

In summary, acetaminophen-related SJS/TEN with SOC was not associated with HLA-class II (HLA-DRB1, DQB1), but we found an association with HLA-B*13:01 and HLA- C*14:03 in addition to HLA-A*02:06 and HLA-B*44:03, which have been described previously.

## Supplementary information

Supplementary Table1

Supplementary Table2
