# Supplementary material for: Association of HLA class I and II gene polymorphisms with acetaminophen-related Stevens–Johnson syndrome with severe ocular complications in Japanese individuals
Source: Hum Genome Var. 2019 Oct 28;6:50. doi: 10.1038/s41439-019-0082-6 (PMC6817890; doi:10.1038/s41439-019-0082-6)
Supplement: Supplementary file 1 — Supplementary Table1 [file 41439_2019_82_MOESM1_ESM.docx]

Supplementary Table 1

Association between HLA-class II and acetaminophen related SJS/TEN with SOC

1. Association between DRB1 and acetaminophen related SJS/TEN with SOC

| HLA-DRB1 | Carrier Frequency | | | | | Gene Frequency | | | | |
| --- | --- | --- | --- | --- | --- | --- | --- | --- | --- | --- |
|  | Case | Control | p-value (Fisher) | corrected-p value | OR | Case | Control | p-value (Fisher) | corrected-p value | OR |
| DRB1*01:01 | 8.8% (7/80) | 9.7% (11/113) | 1.00 |  | 0.9  (0.3-2.4) | 4.4% (7/160) | 4.9% (11/226) | 1.000 |  | 0.9  (0.3-2.4) |
| DRB1*04:03 | 2.5% (2/80) | 5.3% (6/113) | 0.473 |  | 0.5  (0.1-2.3) | 1.3% (2/160) | 3.1% (7/226) | 0.316 |  | 0.4  (0.1-1.9) |
| DRB1*04:05 | 22.5% (18/80) | 24.8% (28/113) | 0.735 |  | 0.9  (0.4-1.7) | 11.3% (18/160) | 13.3% (30/226) | 0.639 |  | 0.8  (0.4-1.5) |
| DRB1*04:06 | 2.5% (2/80) | 6.2% (7/113) | 0.310 |  | 0.4  (0.1-1.9) | 1.3% (2/160) | 3.1% (7/226) | 0.316 |  | 0.4  (0.1-1.9) |
| 3DRB1*08:02 | 7.5% (6/80) | 6.2% (7/113) | 0.775 |  | 1.2  (0.4-3.8) | 3.8% (6/160) | 3.1% (7/226) | 0.779 |  | 1.2  (0.4-3.7) |
| DRB1*08:03 | 27.5% (22/80) | 13.3% (15/113) | **0.0161** | 0.258 | 2.5 (1.2-5.2) | 15.0% (24/160) | 7.1% (16/226) | **0.0168** | 0.167 | 2.3 (1.2-4.5) |
| DRB1*09:01 | 30.0% (24/80) | 31% (35/113) | 1.00 |  | 1.0  (0.5-1.8) | 15.6% (25/160) | 16.8% (38/226) | 0.782 |  | 0.9  (0.5-1.6) |
| DRB1*12:01 | 2.5% (2/80) | 6.2% (7/113) | 0.310 |  | 0.4  (0.1-2.0) | 1.3% (2/160) | 3.1% (7/226) | 0.316 |  | 0.4  (0.1-1.9) |
| DRB1*12:02 | 12.5% (10/80) | 3.5% (4/113) | **0.0239** | 0.382 | 3.9 (1.2-12.9) | 6.3% (10/160) | 1.8% (4/226) | **0.0265** | 0.269 | 3.7 (1.1-12.0) |
| DRB1*13:02 | 22.5% (18/80) | 19.5% (22/113) | 0.719 |  | 1.2  (0.6-2.4) | 11.3% (18/160) | 9.7% (22/226) | 0.735 |  | 1.2  (0.6-2.3) |
| DRB1*14:01 | 5.0% (4/80) | 5.3% (6/113) | 1.00 |  | 0.9  (0.3-3.4) | 2.5% (4/160) | 2.7% (6/226) | 1.00 |  | 0.9  (0.3-3.4) |
| DRB1*14:03 | 5.0% (4/80) | 6.2% (7/113) | 1.00 |  | 0.8  (0.2-2.8) | 2.5% (4/160) | 3.1% (7/226) | 1.00 |  | 0.8  (0.2-2.8) |
| DRB1*14:05 | 2.5% (2/80) | 6.2% (7/113) | 0.310 |  | 0.4  (0.1-1.9) | 1.3% (2/160) | 3.1% (7/226) | 0.316 |  | 0.4  (0.1-1.9) |
| DRB1*15:01 | 13.8% (11/80) | 10.6% (12/113) | 0.509 |  | 1.3  (0.6-3.2) | 6.9% (11/160) | 5.8% (13/226) | 0.673 |  | 1.2  (0.5-2.8) |
| DRB1*15:02 | 11.3% (9/80) | 18.6% (21/113) | 0.226 |  | 0.6  (0.2-1.3) | 5.6% (9/160) | 10.2% (23/226) | 0.135 |  | 0.5  (0.2-1.2) |
| others | 20.0% (16/80) | 18.6% (21/113) | - |  | - | 10.0% (16/160) | 9.3% (21/226) | - |  | - |
| OR, odds ratio; CI, confidence interval.   1. Association between DQB1 and acetaminophen related SJS/TEN with SOC | | | | | | | | | | |
| HLA-DQB1 | Carrier Frequency | | | | | Gene Frequency | | | | |
|  | Case | Control | p-value (Fisher) | corrected-p value | OR | Case | Control | p-value (Fisher) | corrected-p value | OR |
| DQB1*03:01 | 26.3% (21/80) | 21.2% (24/113) | 0.490 |  | 1.3  (0.7-2.6) | 13.8% (22/160) | 10.6% (24/226) | 0.426 |  | 1.3  (0.7-2.5) |
| DQB1*03:02 | 11.3% (9/80) | 17.7% (20/113) | 0.307 |  | 0.6  (0.3-1.4) | 6.3% (10/160) | 9.3% (21/226) | 0.343 |  | 0.7  (0.3-1.4) |
| DQB1*03:03 | 33.8% (27/80) | 35.4% (40/113) | 0.878 |  | 0.9  (0.5-1.7) | 17.5% (28/160) | 19.5% (44/226) | 0.691 |  | 0.9  (0.5-1.5) |
| DQB1*04:01 | 22.5% (18/80) | 23.9% (27/113) | 0.864 |  | 0.9  (0.5-1.8) | 11.3% (18/160) | 12.8% (29/226) | 0.752 |  | 0.9  (0.5-1.6) |
| DQB1*04:02 | 6.3% (5/80) | 5.3% (6/113) | 0.764 |  | 1.2  (0.3-4.0) | 3.1% (5/160) | 2.7% (6/226) | 0.767 |  | 1.2  (0.4-3.9) |
| DQB1*05:01 | 8.8% (7/80) | 9.7% (11/113) | 1.00 |  | 0.9  (0.3-2.4) | 4.4% (7/160) | 5.3% (12/226) | 0.813 |  | 0.8  (0.3-2.1) |
| DQB1*05:02 | 3.8% (3/80) | 8% (9/113) | 0.365 |  | 0.5  (0.1-1.7) | 1.9% (3/160) | 4% (9/226) | 0.373 |  | 0.5  (0.1-1.7) |
| DQB1*05:03 | 7.5% (6/80) | 6.2% (7/113) | 0.775 |  | 1.2  (0.4-3.8) | 3.8% (6/160) | 3.1% (7/226) | 0.779 |  | 1.2  (0.4-3.7) |
| DQB1*06:01 | 35.0% (28/80) | 27.4% (31/113) | 0.271 |  | 1.4  (0.8-2.6) | 19.4% (31/160) | 16.4% (37/226) | 0.498 |  | 1.2  (0.7-2.1) |
| DQB1*06:02 | 13.8% (11/80) | 10.6% (12/113) | 0.509 |  | 1.3  (0.6-3.2) | 6.9% (11/160) | 5.8% (13/226) | 0.673 |  | 1.2  (0.5-2.8) |
| DQB1*06:04 | 21.3% (17/80) | 19.5% (22/113) | 0.856 |  | 1.1  (0.5-2.3) | 10.6% (17/160) | 9.7% (22/226) | 0.864 |  | 1.1  (0.6-2.2) |
| others | 2.5% (2/80) | 1.8% (2/113) | - |  | - | 1.3% (2/160) | 0.9% (2/226) | - |  | - |

OR, odds ratio; CI, confidence interval.
