# Supplementary material for: Association of HLA class I and II gene polymorphisms with acetaminophen-related Stevens–Johnson syndrome with severe ocular complications in Japanese individuals
Source: Hum Genome Var. 2019 Oct 28;6:50. doi: 10.1038/s41439-019-0082-6 (PMC6817890; doi:10.1038/s41439-019-0082-6)
Supplement: Supplementary file 2 — Supplementary Table2 [file 41439_2019_82_MOESM2_ESM.docx]

Supplementary Table 2

Association between HLA-class I and acetaminophen related SJS/TEN with SOC

A. Association between HLA-A and acetaminophen related SJS/TEN with SOC

| HLA-A | Carrier Frequency | | | | | Gene Frequency | | | | |
| --- | --- | --- | --- | --- | --- | --- | --- | --- | --- | --- |
|  | Case | Control | p-value (Fisher) | corrected-p value | OR | Case | Control | p-value (Fisher) | corrected-p value | OR |
| A*01:01 | 0%  (0/80) | 2.03%  (13/639) | 0.380 |  | 0.3*  (0.0-4.9) | 0%  (0/160) | 1.02%  (13/1278) | 0.383 |  | 0.3*  (0.0-4.9) |
| A*02:01 | 25.0% (20/80) | 19.09%  (122/639) | 0.233 |  | 1.4  (0.8-2.4) | 15.6% (25/160) | 10.56%  (135/1278) | 0.0617 |  | 1.6 (1.0-2.5) |
| A*02:06 | 48.8% (39/80) | 13.62%  (87/639) | **4.71E-12** | **6.59E-11** | 6.0 (3.7-9.9) | 27.5% (44/160) | 7.12%  (91/1278) | **8.01E-13** | **1.12E-11** | 4.9 (3.3-7.4) |
| A*02:07 | 7.5% (6/80) | 6.26%  (40/639) | 0.629 |  | 1.2  (0.5-3.0) | 3.8% (6/160) | 3.13%  (40/1278) | 0.634 |  | 1.2 (0.5-2.9) |
| A*03:01 | 0%  (0/80) | 3.60%  (23/639) | 0.0970 |  | 0.2*  (0.0-2.7) | 0%  (0/160) | 1.80%  (23/1278) | 0.0997 |  | 0.2*  (0.0-2.8) |
| A*11:01 | 7.5% (6/80) | 17.21%  (110/639) | **0.0239** | 0.335 | 0.4 (0.2-0.9) | 4.4% (7/160) | 9.31%  (119/1278) | **0.0372** | 0.521 | 0.4 (0.2-1.0) |
| A*24:02 | 43.8% (35/80) | 60.72%  (388/639) | **0.00531** | 0.074 | 0.5 (0.3-0.8) | 23.8% (38/160) | 37.32%  (477/1278) | **6.25E-4** | **8.75E-3** | 0.5 (0.4-0.8) |
| A*24:20 | 1.3%  (1/80) | 1.88%  (12/639) | 1.00 |  | 0.7 (0.1-5.2) | 0.6%  (1/160) | 0.94%  (12/1278) | 1.00 |  | 0.7 (0.1-5.1) |
| A*26:01 | 6.3% (5/80) | 14.40%  (92/639) | 0.0543 |  | 0.4 (0.2-1.0) | 3.1% (5/160) | 7.43%  (95/1278) | **0.0465** | 0.651 | 0.4 (0.2-1.0) |
| A*26:02 | 1.3%  (1/80) | 2.82%  (18/639) | 0.713 |  | 0.4 (0.1-3.3) | 0.6%  (1/160) | 1.49%  (19/1278) | 0.717 |  | 0.4 (0.1-3.1) |
| A*26:03 | 2.5%  (2/80) | 5.95%  (38/639) | 0.300 |  | 0.4 (0.1-1.7) | 1.3%  (2/160) | 2.97%  (38/1278) | 0.307 |  | 0.4 (0.1-1.7) |
| A*31:01 | 12.5% (10/80) | 16.12%  (103/639) | 0.514 |  | 0.7 (0.4-1.5) | 6.3% (10/160) | 8.53%  (109/1278) | 0.365 |  | 0.7 (0.4-1.4) |
| A*33:03 | 25.0% (20/80) | 13.46%  (86/639) | **0.011** | 0.155 | 2.1 (1.2-3.7) | 13.1% (21/160) | 7.12%  (91/1278) | **0.0117** | 0.163 | 2.0 (1.2-3.3) |
| others | 0.0%  (0/80) | 2.50%  (16/639) | - |  | - | 0.0%  (0/160) | 1.25%  (16/1278) | - |  | - |
| OR, odds ratio; CI, confidence interval. * Woolf’s correction  B. Association between HLA-B and acetaminophen related SJS/TEN with SOC | | | | | | | | | | |
| HLA-B | Carrier Frequency | | | | | Gene Frequency | | | | |
|  | Case | Control | p-value (Fisher) | corrected-p value | OR | Case | Control | p-value (Fisher) | corrected-p value | OR |
| B*07:02 | 10.0% (8/80) | 12.05%  (77/639) | 0.715 |  | 0.8 (0.4-1.7) | 5.0% (8/160) | 6.73%  (86/1278) | 0.498 |  | 0.7 (0.3-1.5) |
| B*13:01 | 11.3% (9/80) | 2.97%  (19/639) | **1.98E-3** | **0.0415** | 4.1 (1.8-9.5) | 5.6% (9/160) | 1.49%  (19/1278) | **2.23E-3** | **0.0468** | 3.9 (1.8-8.9) |
| B*15:01 | 5.0% (4/80) | 16.90%  (108/639) | **4.77E-3** | 0.100 | 0.3 (0.1-0.7) | 2.5% (4/160) | 8.61%  (110/1278) | **4.68E-3** | 0.982 | 0.3 (0.1-0.7) |
| B*15:18 | 3.75%  (3/80) | 2.50%  (16/639) | 0.459 |  | 1.5 (0.4-5.3) | 1.9%  (3/160) | 1.25%  (16/1278) | 0.460 |  | 1.5 (0.4-5.2) |
| B*35:01 | 20.0% (16/80) | 15.34%  (98/639) | 0.329 |  | 1.4 (0.8-2.5) | 10.0% (16/160) | 7.75%  (99/1278) | 0.352 |  | 1.3 (0.8-2.3) |
| B*37:01 | 1.3%  (1/80) | 1.72%  (11/639) | 1.00 |  | 0.7 (0.1-5.7) | 0.6%  (1/160) | 0.86%  (11/1278) | 1.00 |  | 0.7 (0.1-5.6) |
| B*39:01 | 12.5% (10/80) | 7.36%  (47/639) | 0.122 |  | 1.8 (0.9-3.7) | 6.3% (10/160) | 3.68%  (47/1278) | 0.130 |  | 1.7 (0.9-3.5) |
| B*40:01 | 7.5% (6/80) | 10.64%  (68/639) | 0.557 |  | 0.7 (0.3-1.6) | 3.8% (6/160) | 5.48%  (70/1278) | 0.454 |  | 0.7 (0.3-1.6) |
| B*40:02 | 13.8% (11/80) | 12.21%  (78/639) | 0.718 |  | 1.1 (0.6-2.3) | 6.9% (11/160) | 6.26%  (80/1278) | 0.731 |  | 1.1 (0.6-2.1) |
| B*40:06 | 5.0% (4/80) | 7.36%  (47/639) | 0.643 |  | 0.7 (0.2-1.9) | 2.5% (4/160) | 3.68%  (47/1278) | 0.649 |  | 0.7 (0.2-1.9) |
| B*44:03 | 30.0% (24/80) | 15.02%  (96/639) | **2.12E-3** | **0.0446** | 2.4 (1.4-4.1) | 15.0% (24/160) | 7.67%  (98/1278) | **3.74E-3** | 0.0784 | 2.1 (1.3-3.4) |
| B*46:01 | 17.5% (14/80) | 8.76%  (56/639) | **0.0251** | 0.527 | 2.2 (1.2-4.2) | 8.8% (14/160) | 4.46%  (57/1278) | **0.0304** | 0.640 | 2.1 (1.1-3.8) |
| B*48:01 | 8.8% (7/80) | 6.26%  (40/639) | 0.346 |  | 1.4 (0.6-3.3) | 4.4% (7/160) | 3.13%  (40/1278) | 0.352 |  | 1.4 (0.6-3.2) |
| B*51:01 | 12.5% (10/80) | 17.68%  (113/639) | 0.274 |  | 0.7 (0.3-1.3) | 8.1% (13/160) | 9.08%  (116/1278) | 0.771 |  | 0.9 (0.5-1.6) |
| B*52:01 | 8.8% (7/80) | 19.87%  (127/639) | **0.0144** | 0.303 | 0.4 (0.2-0.9) | 4.4% (7/160) | 10.02%  (128/1278) | **0.0205** | 0.431 | 0.4 (0.2-0.9) |
| B*54:01 | 7.5% (6/80) | 14.71%  (94/639) | 0.0870 |  | 0.5 (0.2-1.1) | 3.8% (6/160) | 7.75%  (99/1278) | 0.0752 |  | 0.5 (0.2-1.1) |
| B*55:02 | 3.8% (3/80) | 4.69%  (30/639) | 1.00 |  | 0.8 (0.2-2.7) | 1.9% (3/160) | 2.35%  (30/1278) | 1.00 |  | 0.8 (0.2-2.6) |
| B*56:01 | 3.75%  (3/80) | 1.41%  (9/639) | 0.140 |  | 2.7 (0.7-10.3) | 1.9%  (3/160) | 0.70%  (9/1278) | 0.140 |  | 2.7 (0.7-10.1) |
| B*59:01 | 3.75%  (3/80) | 4.07%  (26/639) | 1.00 |  | 0.9 (0.3-3.1) | 1.9%  (3/160) | 2.03%  (26/1278) | 1.00 |  | 0.9 (0.3-3.1) |
| B*67:01 | 0.0%  (0/80) | 2.97%  (19/639) | 0.254 |  | 0.2*  (0.0-3.3) | 0.0%  (0/160) | 1.49%  (19/1278) | 0.257 |  | 0.2*  (0.0-3.3) |
| others | 10.0%  (8/80) | 11.0%  (70/639) | - |  | - | 5.0%  (8/160) | 5.56%  (71/1278) | - |  | - |
| OR, odds ratio; CI, confidence interval. * Woolf’s correction  C. Association between HLA-C and acetaminophen related SJS/TEN with SOC | | | | | | | | | | |
| HLA-C | Carrier Frequency | | | | | Gene Frequency | | | | |
|  | Case | Control | p-value (Fisher) | corrected-p value | OR | Case | Control | p-value (Fisher) | corrected-p value | OR |
| C*01:02 | 31.3% (25/80) | 31.77%  (203/639) | 1.00 |  | 1.0 (0.6-1.6) | 15.6% (25/160) | 17.29%  (221/1278) | 0.657 |  | 0.9 (0.6-1.4) |
| C*03:03 | 22.5% (18/80) | 24.41%  (156/639) | 0.783 |  | 0.9 (0.5-1.6) | 12.5% (20/160) | 12.60%  (161/1278) | 1.00 |  | 1.0 (0.6-1.6) |
| C*03:04 | 32.5% (26/80) | 22.07%  (141/639) | **0.0484** | 0.629 | 1.7 (1.0-2.8) | 16.9% (27/160) | 11.89%  (152/1278) | 0.0759 |  | 1.5 (1.0-2.4) |
| C*04:01 | 10.0% (8/80) | 9.55%  (61/639) | 0.842 |  | 1.1 (0.5-2.3) | 5.0% (8/160) | 4.93%  (63/1278) | 1.00 |  | 1.0 (0.5-2.2) |
| C*06:02 | 1.3%  (1/80) | 2.19%  (14/639) | 1.00 |  | 0.6 (0.1-4.4) | 0.6%  (1/160) | 1.10%  (14/1278) | 1.00 |  | 0.6 (0.1-4.3) |
| C*07:02 | 22.5% (18/80) | 25.98%  (166/639) | 0.587 |  | 0.8 (0.5-1.4) | 12.5% (20/160) | 14.32%  (183/1278) | 0.630 |  | 0.9 (0.5-1.4) |
| C*08:01 | 7.5% (6/80) | 12.21%  (78/639) | 0.269 |  | 0.6 (0.2-1.4) | 3.8% (6/160) | 6.10%  (78/1278) | 0.285 |  | 0.6 (0.3-1.4) |
| C*08:03 | 6.3% (5/80) | 3.13%  (20/639) | 0.184 |  | 2.1 (0.8-5.7) | 3.1% (5/160) | 1.56%  (20/1278) | 0.187 |  | 2.0 (0.8-5.5) |
| C*12:02 | 8.8% (7/80) | 19.87%  (127/639) | **0.0144** | 0.188 | 0.4 (0.2-0.9) | 4.4% (7/160) | 10.02%  (128/1278) | **0.0205** | 0.267 | 0.4 (0.2-0.9) |
| C*14:02 | 11.3% (9/80) | 12.68%  (81/639) | 0.858 |  | 0.9 (0.4-1.8) | 6.3% (10/160) | 6.49%  (83/1278) | 1.00 |  | 1.0 (0.5-1.9) |
| C*14:03 | 28.8% (23/80) | 14.87%  (95/639) | **3.44E-3** | **0.0447** | 2.3 (1.4-3.9) | 14.4% (23/160) | 7.67%  (98/1278) | **6.34E-3** | 0.0825 | 2.0 (1.2-3.3) |
| C*15:02 | 2.5%  (2/80) | 6.89%  (44/639) | 0.152 |  | 0.3 (0.1-1.5) | 1.3%  (2/160) | 3.44%  (44/1278) | 0.227 |  | 0.4 (0.1-1.5) |
| others | 7.5% (6/80) | 5.16%  (33/639) | - |  | - | 3.8% (6/160) | 2.58%  (33/1278) | - |  | - |

OR, odds ratio; CI, confidence interval.
